# Using Polypropylene-Based End-of-Life Vehicle (ELV) Recyclates in Practice: Contaminant and Composition Effects on Performance

**DOI:** 10.3390/polym18192366

**Published:** 2026-09-28

**Authors:** Thomas Lummerstorfer, Markus Gall, Konstanze Kruta, Michael Hettrich-Keller, Dietmar Salaberger, Markus Gahleitner

**Affiliations:** Borouge International Research Center Linz, St. Peterstr. 25, 4021 Linz, Austria; thomas.lummerstorfer@borougeinternational.com (T.L.); markus.gall@borougeinternational.com (M.G.); konstanze.kruta@borougeinternational.com (K.K.); michael.hettrich-keller@borougeinternational.com (M.H.-K.); dietmar.salaberger@borougeinternational.com (D.S.)

**Keywords:** polypropylene, automotive, recycling, mechanics, contamination

## Abstract

Mechanical recycling of polypropylene (PP)-based plastics from end-of-life vehicles (ELVs) is a key route to increase circularity in the automotive sector, but the practical use of ELV recyclates is limited by compositional variability and contamination, especially paint residues. In this study, a PP-based ELV bumper recyclate was compared to a virgin mineral-reinforced and elastomer-modified automotive PP compound and incorporated as a drop-in component at up to 35 wt.-%. Composition was assessed by differential scanning calorimetry, thermogravimetry, and CRYSTEX analysis, while contamination was quantified by computed tomography and related to tensile, impact, and instrumented puncture properties. Mineral and elastomer contents of the ELV recyclate were comparable to the virgin reference after re-stabilization, enabling blends with limited stiffness loss. However, ductility and toughness decreased with increasing recyclate content, especially at low temperature, due to the combined effects of elastomer degradation and rigid contaminant inclusions. Melt filtration of the recyclate substantially reduced the volume fraction of inclusions, removing particularly the largest particles greater than 100 µm, and thereby improved strain at break and puncture performance. Double filtration at 35 wt.-% recyclate loading achieved 50–85% of the virgin-reference ductility and toughness levels and nearly retained the stiffness. These results demonstrate that PP-based ELV recyclates can be used in high-impact automotive formulations at contents exceeding current long-term regulatory targets with recipe modification being limited to further stabilizer addition, provided that contaminant control and elastomer-phase quality are adequately managed.

## 1. Introduction

Every year, over six million automobiles reach the end of their practical use period across Europe, but only a rather small part of the relevant components and materials are recycled systematically. This is especially true for the polymeric components, which make up 14–18 of the mass in average passenger cars in Europe. This equates to about 130–220 kg per vehicle, but due to their density advantage over metal and glass (which is essential for their lifetime benefit in energy consumption) this corresponds to nearly 50% of the total interior and exterior volume [1]. The split by polymer type in Figure 1 shows that about half of this fraction is based on polypropylene (PP), including copolymers and compounds with various elastomers and reinforcements [2,3]. While other types like polyurethane (PU) or polyamide (PA) may be candidates for solvent-based or chemical (hydrolytic) recycling, PP is presently mostly considered for mechanical recycling, including upgrading for re-use in the automotive sector [4].

Efforts in this field need to be intensified, however, as the EU targets for ELV recycling define a target for each vehicle type. These must include recycled plastic, with progressively rising targets: 15% within six years, and 25% within 10 years after entry into force. At least 20% of this recycled content must originate from plastics recycled from end-of-life vehicles or parts removed during the use-phase [5,6]. For designing compositions, however, these limits have to be considered as minimum requirements, as some components are facing additional restrictions for recyclate usage based on emissions (volatiles) or unpainted surface appearance.

In view of these ambitions, the overall situation of plastic recycling in Europe is not really satisfactory, with a total recycling rate of just 26.9% and an average recycled content in new plastic products of 12.6% [7]. The situation is better for packaging-based post-consumer recyclates (PCRs) at 37.8%, compared to 18.8% for the automotive sector. These figures, in combination with a growing polymer fraction in future car constructions, motivated by the trend towards e-mobility, creates a clear need for development in processes and products [8,9].

At the same time, the overall positive effect of polymers on the life cycle analysis (LCA) of modern cars must be considered. This is in line with other application areas both in single- and multiple-use articles, as shown in an extensive study comparing polymers to metals, wood, paper, etc., where an increase in life-cycle greenhouse gas emissions of 35 to 700% was calculated for replacing the respective polymer [10]. For passenger cars, 100 kg of polymers can save 750 L of fuel over a lifespan of 150,000 km, mostly based on weight saving [11,12]. The use of ELV recyclates is further enhancing this positive effect, as advantages of recyclate applications on LCA have been demonstrated independently [13,14].

Most of the literature on mechanical ELV recycling clearly focuses on PP-based materials [15,16,17], although work on high-density polyethylene (HDPE) has been done as well [18]. In only a few studies, however, reaching the requirements of automotive applications again based on these recyclates and thus allowing closed-loop recycling has been attempted by the authors:

- A French study involving Renault compared the performance of three samples of recycled polyolefins from ELVs to be used in automobile applications, specifically as matrix for short glass fiber (SGF)-reinforced compounds [19].
- Direct bumper recycling was attempted at the Wuhan University of Technology (China), including the addition of nano-montmorillonite (nano-MMT), virgin PP, and a compatibilizer [20].
- In our own previous study [4], emulating the performance of a virgin-based bumper compound with PCR and ELV materials was successfully demonstrated.
- A study at the University of Tennessee (USA) dealt with thermal stability and crystallinity of ELV recyclates in relation to virgin materials [21].

For understanding impact modification in multiphase PP systems, a closer look at the actual phase structure—typically including a crystalline PP matrix, elastomeric inclusions and reinforcing particles to achieve the necessary stiffness/impact balance—is necessary. The essential role of advanced PP copolymers in the economical design of automotive compounds has been highlighted before, including the interaction between their molecular structure and morphology [22]. The use of external elastomers as well as reinforcing mineral fillers is established practice. This compound formulation philosophy is reflected in the specific history of PP-based bumpers, which already started in 1978 when the FIAT 127 was the first car with a PP bumper [23]. Later developments expanded the concept of the bumper away from the precursor part made from chromium-plated or painted steel to integrated front-end designs, for which other than mechanics, factors like shrinkage, thermal expansion, surface quality, and paint adhesion are also decisive [24,25,26]. The role of brand owners or original equipment manufacturers (OEMs) in the automotive sector is a dual one here: on the one hand, they need to consider the aforementioned EU targets for ELV recycling; on the other hand, they need to consider customer expectations and can hardly make compromises on product quality and material requirements.

Now, turning to compound formulations with PCR or ELV recyclates as integral components, there is a further need for composition studies of such materials, and the knowledge about their actual origin to have realistic expectations regarding contaminants to be expected [27,28,29]. An elementary issue in ELV recycling is the presence of paint residues, which is aggravated when using bumpers and other exterior components as the main source [30,31,32]. Unless removed prior to mechanical recycling, these residues are present as rigid particles in a size range up to >500 µm in the final part and thus form major defects regarding both mechanics and surface quality.

Various possible ways of handling the paint residue issue have been presented in the literature:

- Selective collection of non-painted parts, like focusing on interior or under-the-hood components [21];
- Automated sorting after shredding [33];
- Removal of paint prior to [34,35] or after shredding [36,37,38,39];
- Melt filtration of the ELV recyclate [31,40].

As references for the present study, the works of Pontes Marques [17] and Puente-Santámaria [35] are specifically relevant, as both present mechanical comparisons for ductility/toughness parameters also tested here.

To better understand the problem, fracture mechanics studies considering particle size distribution of rigid heterogeneities in the polymer compositions were considered. These studies are mostly related to particle size and dispersion quality of mineral reinforcements like talc or calcium carbonate and have a long history. Work has been done for both PP in general, i.e., for single-phase matrices [41,42,43] and for multiphase compositions with crystalline matrix and elastomer inclusions [44,45,46].

Common to the results of all these studies is that other than the particle size and dispersion quality, with the principle of biggest particles dominating failure initiation, in line with stress concentration being proportional to the square root of dimension [41,43], matrix adhesion and internal cohesion also play important roles. Toughness losses by too big or badly dispersed reinforcements are not limited by the presence of elastomer particles, although the final property balance is better [46]. The approach of embedding rigid particles in an elastomeric shell helps here, but also limits the stiffening effect of the reinforcement [45], and there is no way to prevent internal failure of aggregates or particles with limited cohesion like wood fibers [47]. An early study on effects of paint particles confirms the similarity of these regarding toughness limitation [48], but in practice paint particles have been found to be more difficult to break up or remove by melt filtration due to their plasticity at elevated temperatures.

The scope of the study was to use an ELV recyclate having a composition comparable to a virgin PP bumper compound so that it can be used as a simple “drop-in” to modify the same. In varying both concentration and possible pre-treatment steps, an understanding of the effects of contaminant particles (mostly paint) and the respective possibilities to reduce them by melt filtration should be achieved. At the same time, detailed composition differences between ELV recyclate and virgin materials were studied to get a realistic upper limit for its concentration in the final application, based on OEM requirements.

## 2. Materials and Methods

A mineral-reinforced and elastomer-modified high-performance polypropylene compound intended for injection-molded automotive parts [49,50] was used as virgin reference and designated PPB-V. ELV recyclate obtained as shredded flakes from bumper recycling in Germany was prepared internally prior to further characterization and compounding. The irregularly shaped flakes (see Figure 2) were pelletized using a recycling single-screw extruder equipped with a continuously self-cleaning 100 µm melt filter to remove impurities such as paint and metal residues. In this compounding step, 0.30 wt.-% of a 1:1 mixture of pentaerythrityl-tetrakis(3-(3′,5′-di-tert. Butyl-4-hydroxyphenyl)-propionate (Irganox 1010 from BASF SE, Ludwigshafen, Germany) and Tris (2,4-di-t-butylphenyl) phosphite (Irgafos 168 from BASF SE, Germany) were added to re-stabilize the material. This re-stabilization with a combination of primary and secondary antioxidants is clearly recommended based on the longer usage period of ELV material in relation to PCR and has been found to be positive in both practice and the literature [51,52].

This standard recyclate was designated PPB-ELV-100, while a version without filtration is PPB-ELV-0. An additional version with double filtration using a 100 µm melt filter followed by an additional finer melt filtration step using a 50 µm screen, was designated PPB-ELV-50. The same batch of primary material was used both for the ELV recyclate from the external source and the virgin reference from our own standard production, being well-aware of the typical issues with batch-to-batch variation, which is more severe in recycled polymers.

The virgin reference PPB-V and the standard recyclate PPB-ELV-100 were characterized in analogy to our earlier study [4] as follows (see Table 1 for results):

- Melt flow rate (MFR) was measured according to ISO 1133 [53] at 230 °C and 2.16 kg load (precision < +/−10% relative).
- Differential scanning calorimetry (DSC) was run according to ISO 11357/part 3/method C2 [54] in a heat/cool/heat cycle with a scan rate of 10 °C/min in the temperature range of −30 to +225 °C with a TA Instruments Q200 (TA Instruments, New Castle, DE, USA) on 5 to 7 mg samples. The two melting temperatures corresponding to the PE (T_m,PE_) and PP content (T_m,PP_) and the two corresponding melting enthalpies (H_m,PE_) and (H_m,PP_) were determined from the second heating step (single-point measurements).
- Thermogravimetry (TGA) was used to determine the inorganic content of the virgin and PCR materials according to ISO 1172:2023 [55] with a Perkin Elmer (Shelton, CT, USA) TGA 8000. Approximately 10–20 mg of material was placed in a platinum pan, temperature was equilibrated at 50 °C for 10 min, and afterwards raised to 950 °C under nitrogen at a heating rate of 20 °C/min. The ash content was evaluated as the wt.-% at 850 °C (single-point measurement).

The CRYSTEX method [29,56] was used to determine the crystalline (CF) and soluble (SF) fractions and their respective properties (intrinsic viscosity, IV, and ethylene content, C2). A Crystex QC apparatus of PolymerChar (Valencia, Spain) was used for this analysis, which is an excellent alternative to the frequently used separation of PP copolymers and compositions into xylene cold soluble and insoluble fractions (XCS/XCI) followed by analysis of the fractions. CF and SF are separated through temperature cycles of dissolution at 160 °C, crystallization at 40 °C and re-dissolution in 1,2,4-trichlorobenzene at 160 °C. Quantification of SF, CF, and ethylene content (C2) are achieved by means of an integrated infrared detector (IR4) and for the determination of the intrinsic viscosity (IV) an online 2-capillary viscometer is used (single-point measurements).

The two compositions were thus found to be sufficiently similar in most relevant composition parameters like mineral (ash) content, elastomer content (SF), and overall molecular weight, indicated by MFR and IV(total), to be used for a blending study. Even the elastomer composition expressed by its C2 content and molecular weight (C2(SF) and IV(SF), resp.) is similar with respect to its typical effect on mechanics as shown for ICPs in general [22,57].

For the blending study, the virgin reference PPB-V was melt-mixed with increasing amounts of the homogenized and filtered bumper recyclate PPB-ELV-100, generating compositions ranging from 0 wt.-% (pure virgin reference) to 35 wt.-% recyclate content. These blends were produced using a co-rotating twin screw extruder ZSE 27 of Leistritz Extrusionstechnik GmbH, Nürnberg, Germany, equipped with a typical screw configuration for PP-based automotive compounds. A screw speed of 600 rpm, a melt temperature of 210 °C, and a throughput of 90 kg/h was applied. No additional melt filtration was applied in this step. At 35 wt.-% recyclate content, additional compounds were prepared using either non-filtered PPB-ELV-0 or the more intensively filtered PPB-ELV-50 version.

For characterization, other than MFR, ash content, and CRYSTEX as defined above, the amount and size of defects (paint residues and mineral aggregates) was also determined by computed tomography (CT) as described before [28,58]. Cut-outs of 60 × 60 × 3 mm^3^ injection-molded plaques were scanned with a resolution of 5 µm Voxel edge length. The smallest particles that were taken into account were ca. 15 µm in length. Earlier specialized studies [59,60] have determined a probability of detection (POD) for this. However, as a large fraction of the particles was found to be above 100 µm, the POD for smaller ones was not determined, considering that this part of the distribution is less relevant for crack initiation [41,43].

The scans were performed using a Thermo Fisher Scientific Heliscan device. 3D data analysis including segmentation and determination of features of all segmented inclusions was done using the software Avizo (Thermo Fisher Scientific, Linz, Austria). Since the contrast between painted inclusions and the polymer matrix was high, a simple manual threshold was applied to separate these particles. Fibers, most likely being glass fibers, and particles, predominantly being paint residues, were quantified collectively and separately, with a separate class for particles above a maximum length of 100 µm. Identity of the majority of the inclusions in the paint compositions was ascertained by infrared microscopy on multiple particles in relation to an earlier patent application, in which details can be found [58]. The PPB-V contains a fine talc type with a d95 (i.e., 95% of the particle below that value) of 3.5 µm [22], meaning that the CT method cannot resolve it.

In addition, a full mechanical characterization of the references and all blends was performed on injection-molded specimens, testing 12 specimens in each case and averaging over the 10 results closest to average:

- Tensile tests for determining modulus, strain and stress at break, were performed according to ISO 527-2 [61] at +23 °C with a cross head speed for modulus 1 mm/min and a general test speed 50 mm/min using injection-molded specimens 1B prepared as described in ISO 1873-2 with a melt temperature of 230 °C, mold temperature of 40 °C, injection speed of 0.2 m/s, holding time 40 s and overall cycle time 60 s (dog bone shape, 4 mm thickness [62]). Precision is different for the various parameters, with < +/−5% relative for modulus and stress at break but < +/−10% relative for strain at break.
- Charpy notched impact strength (NIS) was measured according to ISO 179 1eA (precision < +/−10% relative) [63] at +23 and −20 °C on test bars of 80 × 10 × 4 mm^3^ injection-molded according to ISO 1873-2 (same parameters as above) [59].
- Instrumented puncture tests (IPTs) were done in accordance with ISO 6603-2 (precision < +/−10% relative) [64] at +23 and −30 °C on injection-molded plaques of 60 × 60 × 3 mm^3^, reporting the puncture (E_punct_) and penetration energy (E_max_).

The respective compositions and characteristics of the resulting compounds can be found in Table 2. Compositions at 35 wt.-% recyclate with different melt filtration settings (non-filtered PPB-ELV-0, more intensively filtered PPB-ELV-50) are reported further below. It should be noted that particles as small as the talc filler used in the PPB-V will not be visible in CT and are not part of the analysis results.

It should be noted that the mechanical performance of the filtered and homogenized PPB-ELV-100 is already significantly improved regarding ductility (strain at break) and toughness (impact and penetration strength) over PPB-ELV-0, as can be seen in Figure 3, which also includes data for the virgin grade PPB-V. Based on the filtration effects discussed below one can assume that the non-filtered ELV recyclate—which would be difficult to quantify representatively by CT—has an even higher level of contamination, and especially a higher fraction of big inclusions (>100 µm).

## 3. Results and Discussion

The large similarity in overall composition between the base materials of the main blending series, PPB-V and PPB-ELV-100, makes the results particularly interesting. In it, the two parameters commonly considered most relevant for mechanical performance of a mineral-reinforced PP impact copolymer composition [23,45,46] remain constant or are just varied in a small range. The mineral filler content, predominantly talc as verified before [4], stays on the same level between 14.6 and 13.2 wt.-%, although the talc type(s) and size distribution are unknown for the recyclate and can certainly affect the performance too [42,44]. The SF content also just varies between 34.0 and 30.4 wt.-%, but again composition of the elastomer phase will be different (C2(SF) 52.8 vs. 50.7 wt.-% and IV(SF) 2.43 vs. 2.12 dL/g). Without knowing the initial properties, it is still clear that re-stabilization of ELV material cannot compensate for damage from the usage period and earlier processing [51].

As the SF, i.e., amorphous elastomer content, is even lower for the pure PPB-ELV-100, the variation in stiffness, as expressed by the tensile modulus in Figure 4a, must result from matrix crystallinity or PE content differences. The latter is limited again, as evidenced by the small PE contribution in DSC, but lower Tm and Tc for the recyclate indicate presence of random copolymers and possibly degradation in the matrix-PP, both reducing crystallinity and stiffness. The former factor is well-documented [65], but for degradation in mechanical recycling, divergent results with both increasing [66] and decreasing crystallinity [67] have been reported. The latter of these two studies seems more realistic, however, as it deals with actual recycled (PCR) samples and not just re-extruded virgin material.

The effect of recyclate addition on ductility at 23 °C as expressed by the strain at break in Figure 4b is already less linear. Addition of 5 to 10 wt.-% PPB-ELV-100 already results in a sizeable drop, and at 35 wt.-% ductility is reduced by about 30%. Despite the fact that contaminants make up a volume fraction of less than 1‰ after filtration, their increasing number may be partly responsible here, and equally for the even more drastic reduction in low-temperature toughness (see Figure 5). When drawing parallels to earlier compound studies, predominant effects of small fractions of the biggest particles have been observed for both talc [41,44] and wood fibers [47]. For paint particles, only limited data are available so far [48], but neither the adhesion to the polymer matrix nor the internal cohesion can be expected to be good, making them rather ideal defects for crack initiation.

The fact that the toughness loss still increases significantly between −20 and −30 °C, however, indicates that elastomer structure and matrix–elastomer interaction may be partly responsible as well. Understanding these effects requires a closer look at impact copolymer design. Other than the amount of elastomer ethylene-propylene copolymer (EPC [68]), its molecular weight as represented by the intrinsic viscosity (like IV(SF)) or rather its relation to the matrix molecular weight is also a decisive factor for phase morphology and impact strength. In a series with constant matrix viscosity and EPC composition, the IV-ratio (like IV(SF)/IV(CF)) could be shown to have an optimum range around 2 for toughness [69], while other parameters like surface quality and shrinkage require even higher levels [26] and transparency is best at lower ones. In the present study, increasing the ELV recyclate content gradually reduces the IV ratio and even more so the absolute IV(SF) itself, both likely contributing to impact strength reduction.

An important difference between PCR and ELV material must be considered here: the former typically has less elastomer content, and that elastomer is often of a minor quality, both reducing the extent to which it can be used for high-impact automotive applications or in consequence requiring additional external elastomer in the final formulation. The ELV material, on the other hand, has a much longer lifetime and is exposed to harsher environmental conditions. Degradation of multiphase high-impact PP is generally complex, as the EPC phase will react differently to radical attacks than the matrix [70], changing the IV ratio and sometimes even generating crosslinked gels. In real mechanical recycling, especially after an extended usage period, this effect can be even more complex [71], especially in interaction with PE [72].

The second decisive factor is the comonomer content of the EPC (like C2(SF)) which defines the glass transition temperature of this phase [22]. For reactor grades based on Ziegler–Natta-type catalysts, there is a reasonable upper limit around 50 wt.-%, as beyond that crystalline EPC appears in significant amounts, and crystalline PE might even form. This affects compatibility to the matrix and, again, phase morphology [73]. The crystalline PE will affect some parameters like stress whitening positively, while low temperature behavior will deteriorate. These principles also apply when combining reactor-made with externally added elastomers [74], meaning that the averages determined by CRYSTEX for the compositions in this study are also helpful for understanding the effect (see Figure 6b). In the present study, C2(SF) is always rather high, but the higher C2 content in the crystalline fraction (9.4 vs. 4.2 wt.-% for the virgin reference) is unlikely to stem from crystalline PE, as that would show up in the DSC as well. The lower modulus and T_m_ discussed before rather suggest presence of a random copolymer fraction.

While changing the elastomer composition and molecular weight relation of the ELV recyclate is beyond the toolbox of compounding, reducing the amount of (at least bigger) contaminants can be done by melt filtration of the starting material. As already seen in Figure 3 for the primary filtration of the PPB-ELV-0, this can at least restore some of the ductility and toughness of the recyclate, and the data in Table 3 also confirm its efficiency for compounding. This comparison was done at an ELV recyclate content of 35 wt.-%, which is well above the overall EU target explained above, meaning that components from such compositions can contribute positively to the overall balance.

The filtration effect on contamination is evident in the CT results, with Figure 7 presenting a direct comparison. Both particles and (glass) fibers are clearly visible in all three images, and the drop by about 50% is reflected by the apparent particle density difference between the non-filtered reference PPB-B35-0 and the two filtered versions, which among each other show much less difference, however. Even reduction in the biggest particles (>100 µm) achieved in filtration is ~66%, and the rather limited effect of a second, finer filtration is also obvious here.

The mechanical consequences are presented in Figure 8. For all toughness and ductility parameters, 50–85% of the target (reference) level are achieved by double filtration, and nearly 95% for the stiffness, which is already close to the confidence interval given by the respective ISO standard of +/−5% (see Figure 8). This confirms the dominant effect of paint residues as contaminants on all fracture phenomena, be it under quasi-static tensile load at room temperature or under multi-axial impact load at −30 °C. This, however, also shows the limits of melt filtration, as the second and finer step (which causes higher cost and bears the risk of additional polymer damage) only resulted in rather moderate performance improvement.

## 4. Summary and Conclusions

Based on the considerations presented in the introduction, there is a clear need for increasing the recycling rate of ELV materials while also maintaining the performance of automotive materials based on PP. In our previous study [4], the advantages of ELV recyclate over PCR material were shown for optimized compositions, balancing the differences with variations in elastomer and filler addition while increasing recyclate content from 25 to 40%. The present work aimed at “drop-in” replacement of an optimized compound at intermediate recyclate contents, trying to limit recipe modification to stabilizer addition as recommended in the literature [51,52]. A good compromise was found at 35 wt.-%, which is well above the overall long-term EU target of 25%, meaning that the respective components will leave room for more difficult parts with limitations regarding emissions or surface quality.

While the same batch was used both for the ELV recyclate (PPB-ELV-100 from the external source) and the virgin reference (PPB-V from own standard production) to avoid the issue of batch-to-batch variation, the results must be considered with care. From our perspective, it is obvious that proper quality control of the incoming recyclate is a necessary condition for successful production of ELV-containing materials.

Paint residues appeared as one particularly critical factor for compound performance, especially for impact strength and ductility. Melt filtration was found to be a suitable tool for improvement here. Figure 9 presents a rather qualitative comparison between the results of the present study and earlier work related to the presence and removal of paint residues [17,35]. This cannot be a direct comparison, however, as the compositions and stiffness-impact balances of the references are quite different:

- The composition in the present study has an elastomer content of ~34 wt.-% with little PE, a tensile modulus of ~1700 MPa and Charpy NIS (23 °C) of ~35 kJ/m^2^.
- The material of the 2024 study is rather comparable in stiffness at an original Charpy level of ~22 kJ/m^2^.
- The ELV material in the most recent study has a high PE fraction (~27% by DSC) and a much lower stiffness of ~1000 MPa.

Obviously, the range of potential improvement in impact performance and ductility is rather wide, depending on the selected pairing of ELV feedstock and treatment applied. A more systematic understanding of polymer composition and contamination effects is necessary for increasing ELV content in a sustainable way without requiring additional (and costly) modification [20,75]. One obvious aspect is the retention of elastomer quality over extended usage period, as seen by parameters like IV(SF), which is known to be critical for impact strength from ICP development for many years already [67,73]. Another aspect is the presence and, very likely, also the size distribution of paint residues, where clearly a more systematic comparison of different paint removal processes for one given composition would be needed.

## Figures and Tables

**Figure 1:**
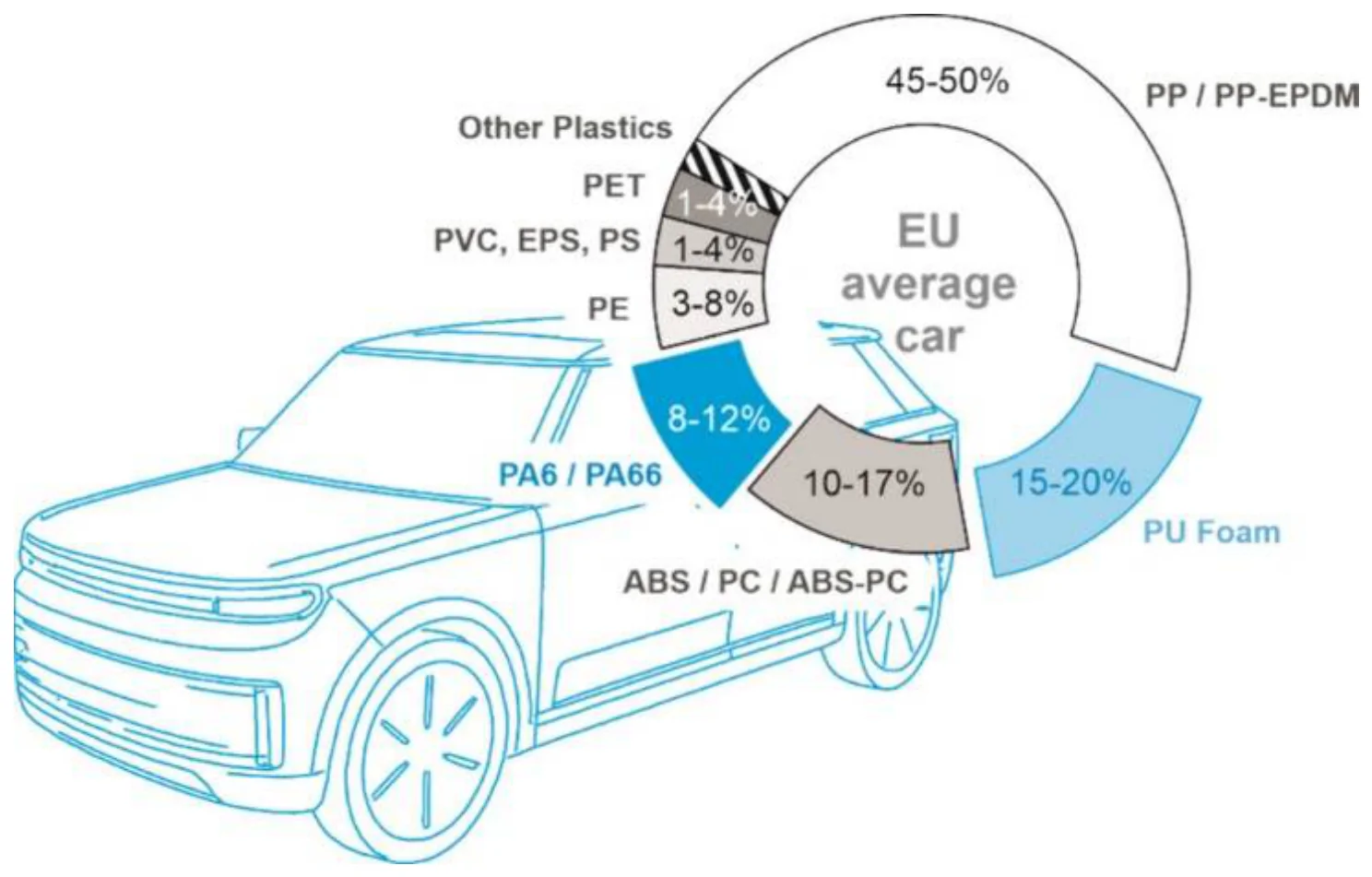
Composition of the polymeric fraction of an average European passenger car by polymer type (reprinted from Von Vacano et al., Accounts of Materials Research 2026 [3], reproduced under Creative Commons License).

**Figure 2:**
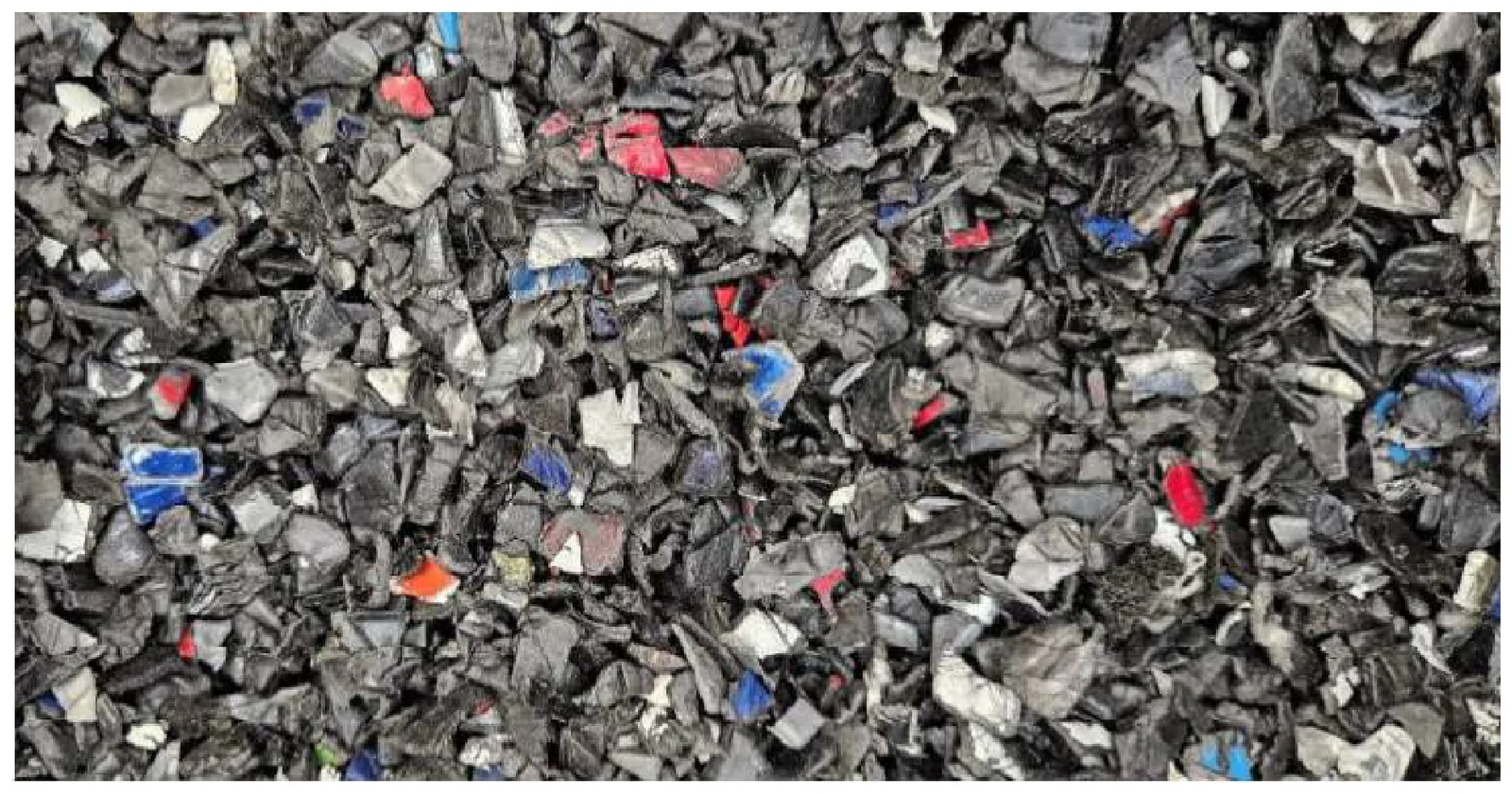
Optical appearance of ELV bumper flakes as received (image height 10 cm).

**Figure 3:**
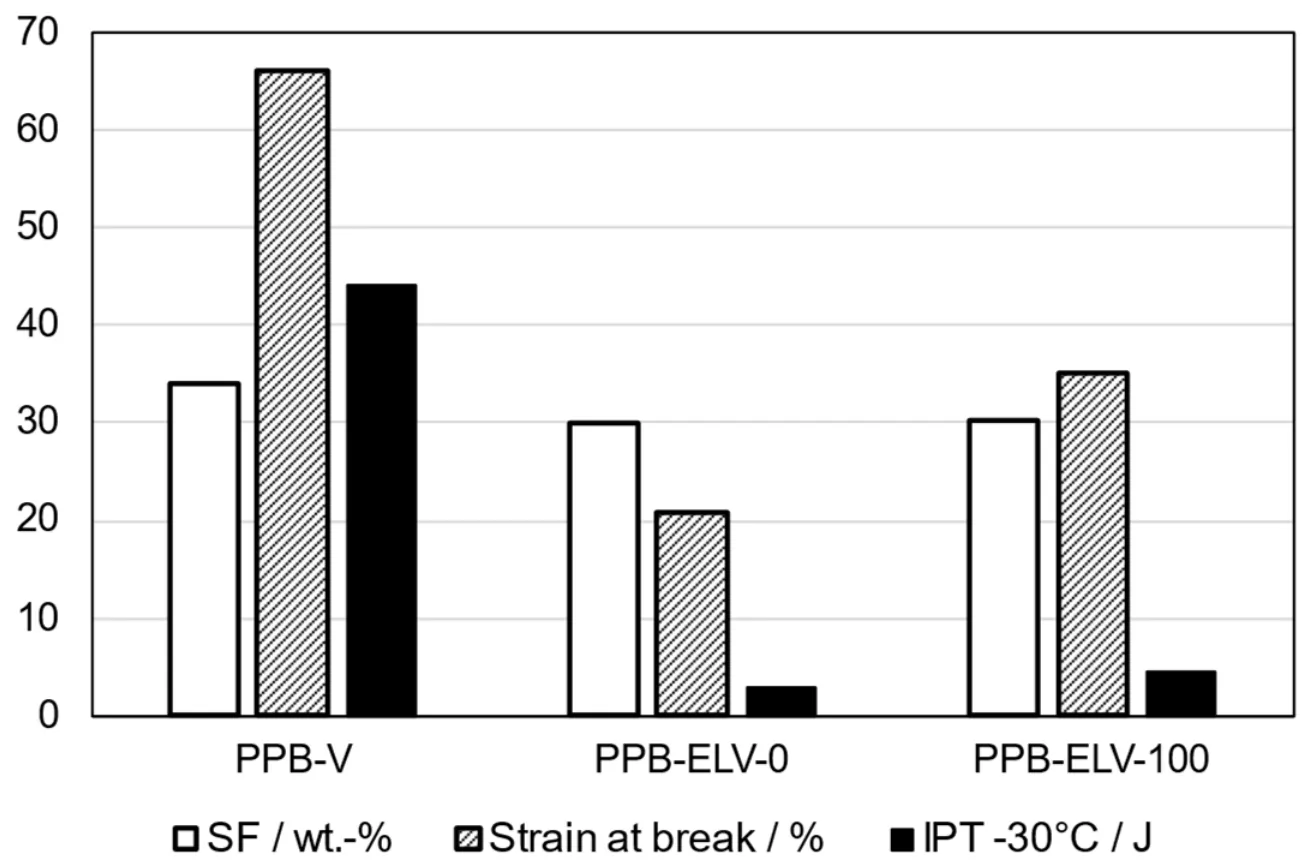
Comparison of elastomer (SF) content, ductility (strain at break at 23 °C) and low-temperature toughness (instrumented puncture at −30 °C) between virgin reference, non-filtered and standard (100 µm) filtered ELV recyclate.

**Figure 4:**
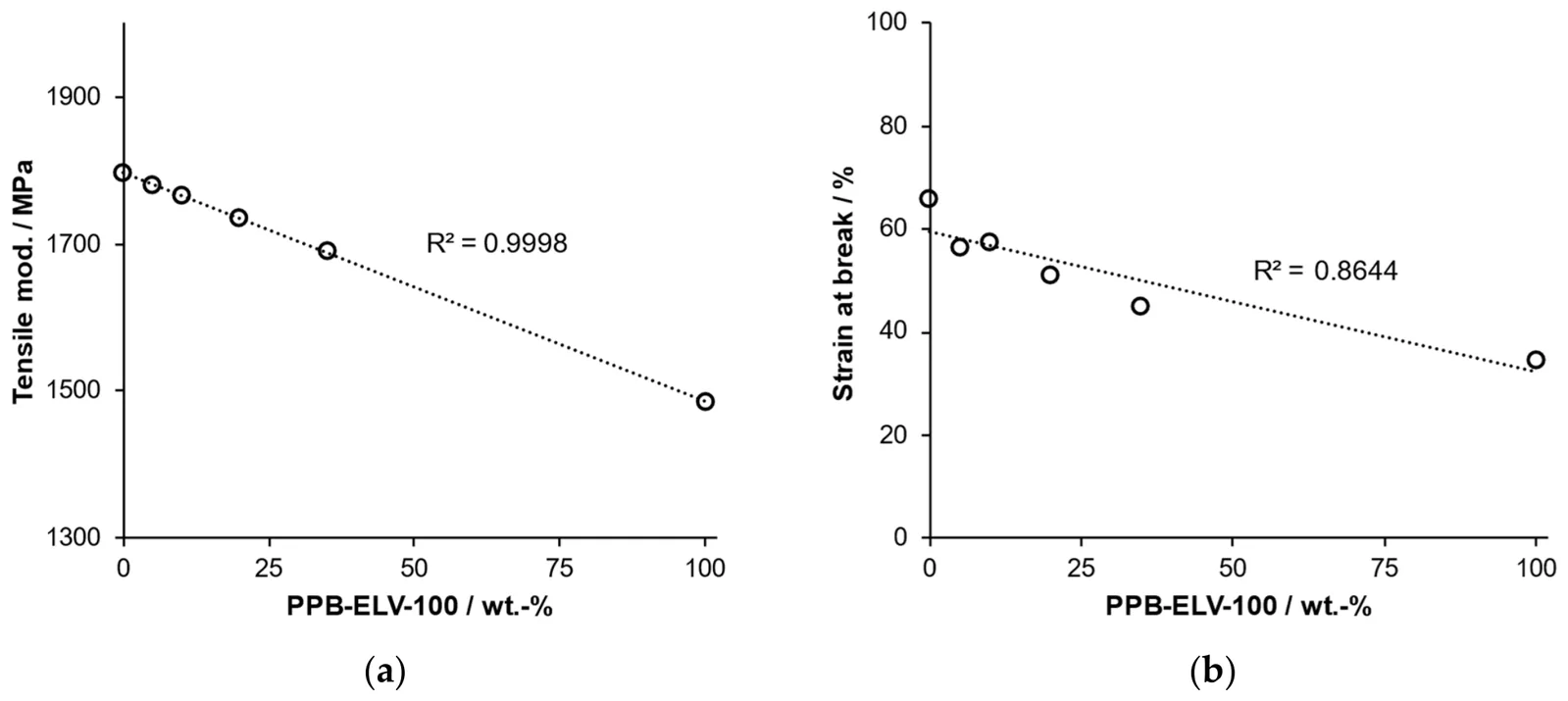
Tensile properties of the main blending series as function of recyclate content; (**a**)—Tensile modulus; (**b**)—Strain at break.

**Figure 5:**
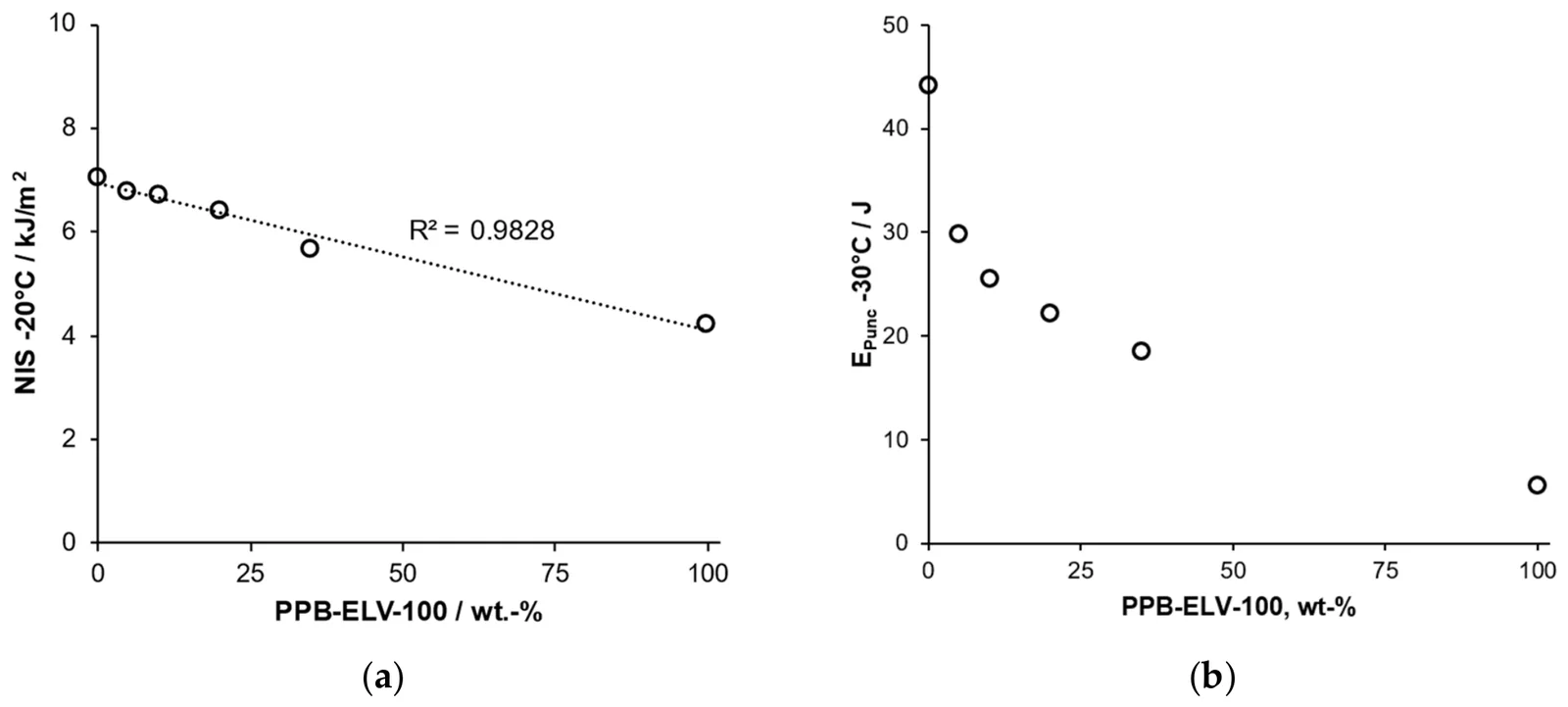
Low-temperature toughness of the main blending series as function of recyclate content; (**a**)—Charpy NIS −20 °C; (**b**)—Puncture energy IPT −30 °C.

**Figure 6:**
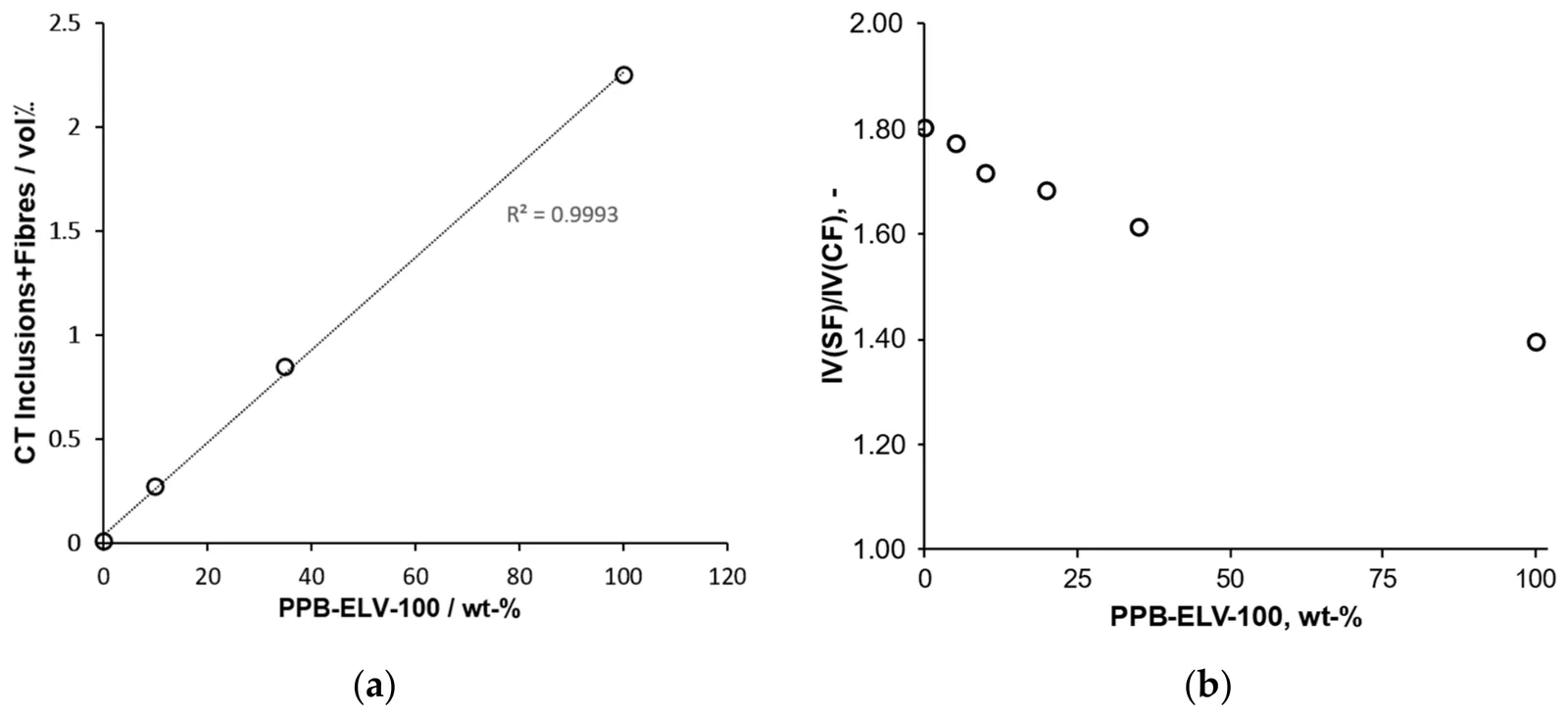
Effect of increasing recyclate content on impact-critical parameters of the main blending series; (**a**)—CT inclusions total; (**b**)—IV-ratio between elastomer and matrix fraction.

**Figure 7:**
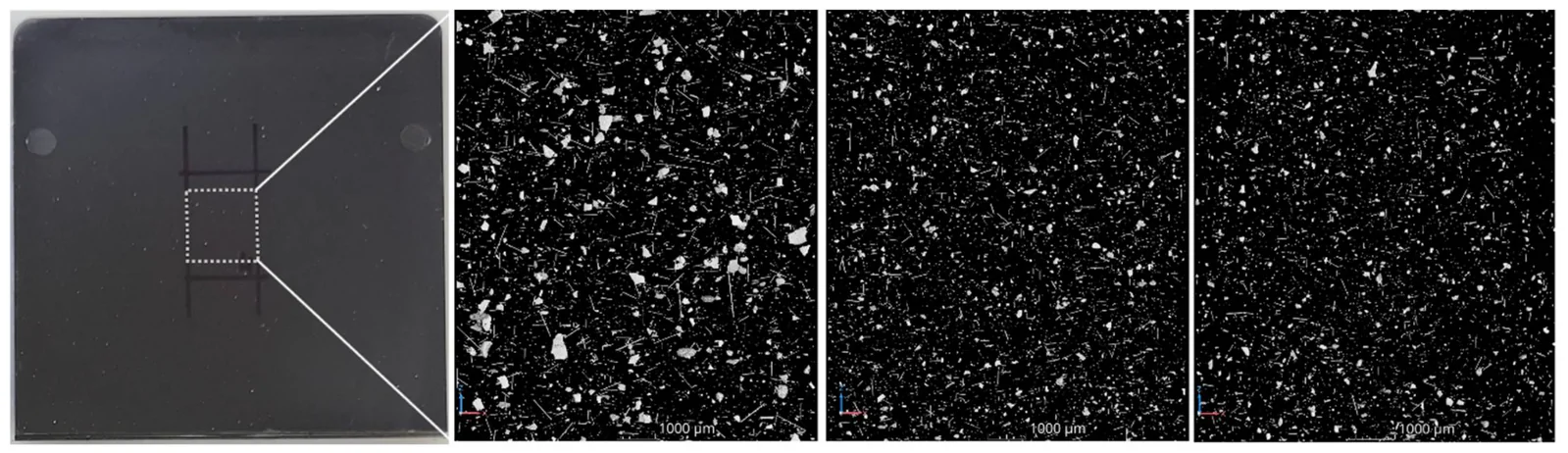
CT images of 35 wt.-% recyclate blends, differently filtered, left to right: Sample segment on plaque, PPB-B35-0 (not filtered), PPB-B35 (normal filtration) and PPB-B35-50 (double filtration).

**Figure 8:**
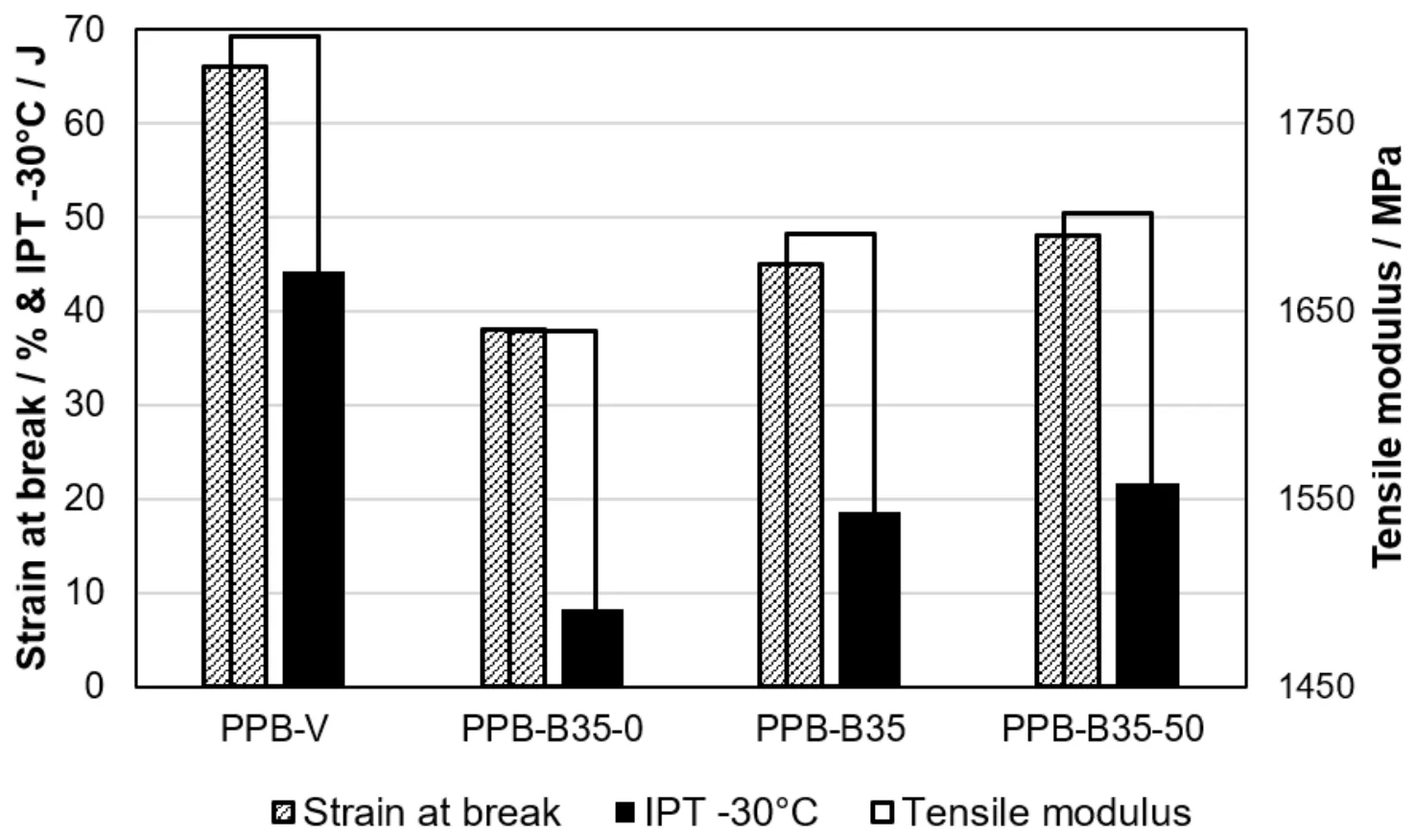
Melt filtration effect at an ELV recyclate content of 35 wt.-% comparing stiffness (tensile modulus), ductility (strain at break 23 °C) and low-temperature toughness (instrumented puncture −30 °C) to levels of virgin reference.

**Figure 9:**
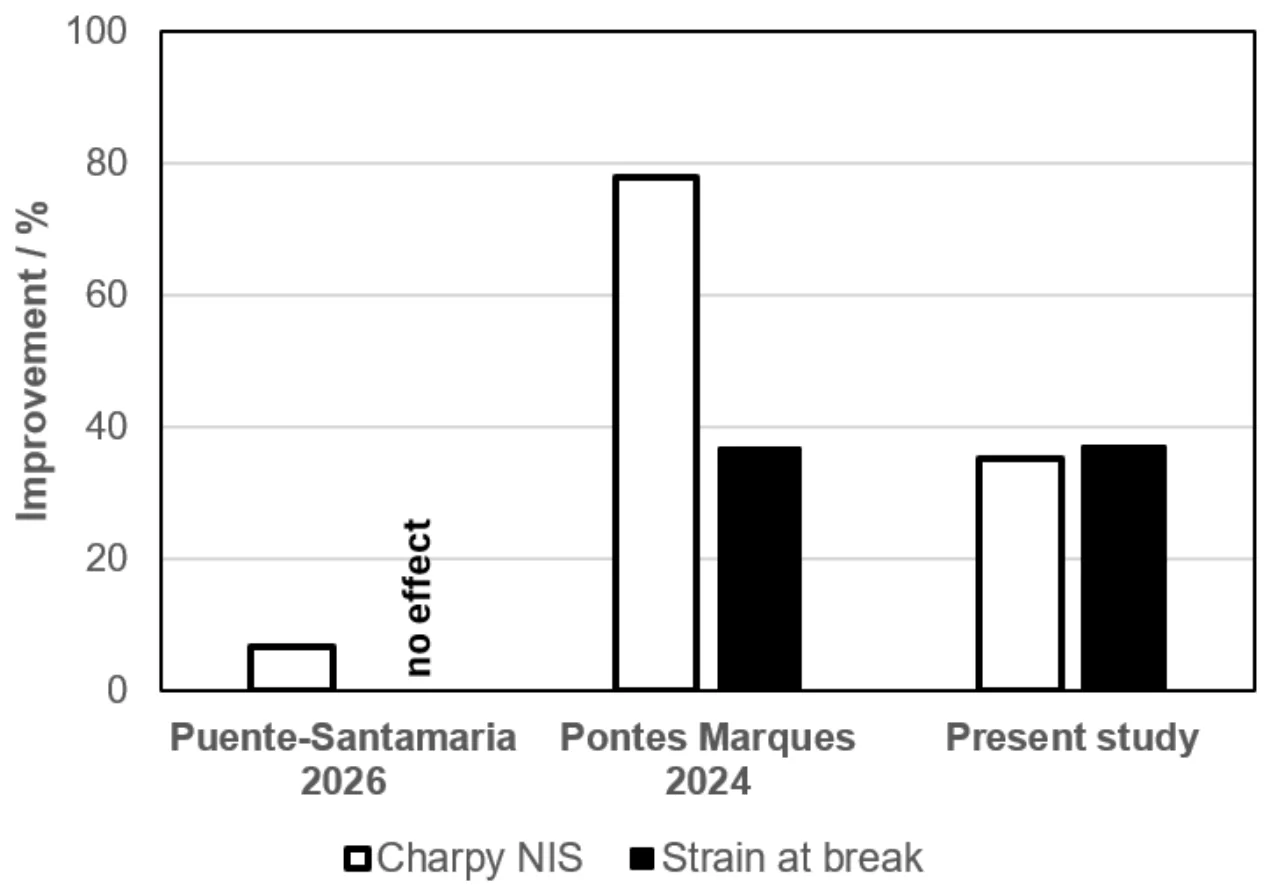
Qualitative improvements in impact and ductility compared to paint removal treatments in earlier studies (Puente-Santamaría 2026 [35]: Untreated recyclate vs. parts with 60 min caustic wash; Pontes Marques 2024 [17]: Painted vs. unpainted parts, both compounded and injection-molded; Present study: PPB-ELV100 vs. PPB-B35-50).

**Table 1:** Composition analysis results of virgin reference and ELV recyclate (*—230 °C/2.16 kg; ** TGA residue at 850 °C; *** IV(SF)/IV(CF)).

		PPB-V	PPB-ELV-100
MFR *	g/10 min	24.2	14.3
Ash **	wt.-%	14.6	13.2
**DSC**			
Tm(PE)	°C	103.6	124.5
Hm(PE)	J/g	0.4	1.0
Tm(PP)	°C	165.2	164.7
Hm(PP)	J/g	65.6	68.8
Tc	°C	129.4	127.4
**CRYSTEX**			
C2(total)	wt.-%	19.6	21.4
IV(total)	dL/g	1.74	1.72
SF	wt.-%	34.0	30.4
C2(SF)	wt.-%	52.8	50.7
IV(SF)	dL/g	2.43	2.12
CF	wt.-%	66.0	69.6
C2(CF)	wt.-%	4.1	9.4
IV(CF)	dL/g	1.35	1.52
IV-ratio ***	-	1.80	1.39

**Table 2:** Characterization results of blending series (*—230 °C/2.16 kg; ** TGA residue at 850 °C; *** IV(SF)/IV(CF); n.d.—not determined).

		PPB-V	PPB-B5	PPB-B10	PPB-B20	PPB-B35	PPB-ELV-100
PPB-V	wt.-%	100	95	90	80	65	0
PPB-ELV-100	wt.-%	0	5	10	20	35	100
MFR *	g/10 min	24.2	23.5	22.9	21.6	20.0	14.6
Ash **	wt.-%	14.6	14.8	14.6	14.4	13.8	13.2
CRYSTEX							
C2(total)	wt.-%	19.6	19.3	19.8	19.4	19.2	21.4
SF	wt.-%	34.0	33.8	33.7	33.2	32.7	30.4
C2(SF)	wt.-%	52.8	52.6	52.5	52.4	52.0	50.7
C2(CF)	wt.-%	4.2	4.4	4.4	4.7	4.9	9.4
IV(SF)	dL/g	2.43	2.41	2.35	2.34	2.29	2.12
IV-ratio ***	-	1.80	1.72	1.71	1.68	1.61	1.44
CT results							
CT total	vol.- ‰	0.01	n.d.	0.27	n.d.	0.85	2.90
CT particles	vol.- ‰	0.00	n.d.	0.23	n.d.	0.67	2.25
CT > 100 µm	vol.- ‰	0.00	n.d.	0.12	n.d.	0.38	1.65
Mechanics							
Tensile Mod.	MPa	1796	1781	1766	1735	1691	1485
Stress at break	MPa	12.7	12.7	12.7	12.7	12.9	13.2
Strain at break	%	66	56	57	51	45	35
NIS +23 °C	kJ/m^2^	50.3	43.6	43.6	38.6	35.1	25.6
NIS −20 °C	kJ/m^2^	7.0	6.8	6.7	6.4	5.7	4.2
IPT +23 °C E_max_	J	18.5	18.7	19.0	18.2	18.2	17.4
IPT +23 °C E_punct_	J	34.3	34.3	33.7	31.7	31.1	23.0
IPT −30 °C E_max_	J	30.0	27.7	23.9	20.7	17.2	3.6
IPT −30 °C E_punct_	J	44.2	29.9	25.5	22.2	18.6	4.4

**Table 3:** Filtration effects for blends with 35 wt.-% recyclate (*—230 °C/2.16 kg; ** TGA residue at 850 °C).

		PPB-B35-0	PPB-B35	PPB-B35-50
Base	65 wt.-%	PPB-V	PPB-V	PPB-V
ELV	35 wt.-%	PPB-ELV-0	PPB-ELV-100	PPB-ELV-50
MFR *	g/10 min	19.7	20.0	19.9
Ash **	wt.-%	14.1	13.8	14.0
CT total	vol.- ‰	1.61	0.85	0.82
CT particles	vol.- ‰	1.34	0.67	0.67
CT > 100 µm	vol.- ‰	1.15	0.38	0.40
Mechanics				
Tensile Mod.	MPa	1639	1691	1702
Stress at break	MPa	12.6	12.9	12.7
Strain at break	%	38	45	48
NIS +23 °C	kJ/m^2^	32.7	35.1	34.6
NIS −20 °C	kJ/m^2^	6.1	5.7	6.0
IPT +23 °C E_max_	J	18.4	18.2	18.7
IPT +23 °C E_punct_	J	25.8	31.1	30.3
IPT −30 °C E_max_	J	7.2	17.6	20.2
IPT −30 °C E_punct_	J	8.2	18.6	21.7

## Data Availability

The original contributions presented in this study are included in the article. Further inquiries can be directed to the corresponding author.

## Abbreviations

The following abbreviations are used in this manuscript:

C2	Ethylene
CF	Crystalline fraction (CRYSTEX analysis)
CT	Computed tomography
DSC	Differential scanning calorimetry
ELV	End-of-life vehicles
EPC	Ethylene-propylene copolymer
EU	European Union
ICP	Impact copolymer (multiphase polypropylene composition)
IPT	Instrumented puncture test
IV	Intrinsic viscosity
LCA	Life cycle analysis
MFR	Melt flow rate
NIS	Notched impact strength (Charpy)
OEM	Original equipment manufacturer
PA	Polyamide
PCR	Post-consumer recyclate (packaging)
PE	Polyethylene
PP	Polypropylene (isotactic)
PU	Polyurethane
SGF	Short glass fiber
SF	Soluble fraction (CRYSTEX analysis)
TGA	Thermogravimetric analysis
